# Nanoscale dipole dynamics of protein membranes studied by broadband dielectric microscopy†
†Electronic supplementary information (ESI) available: Bacteria rhodopsin – structure and dipole moments, visualization of surface water from topography images and force distance curves, dielectric spectra on the purple membrane: raw data, finite element modelling for quantification of measurements, the dielectric spectrum with an extended frequency range, and comparison of dielectric spectra on a monolayer purple membrane at three values of the relative humidity. See DOI: 10.1039/c8nr05880f


**DOI:** 10.1039/c8nr05880f

**Published:** 2019-02-05

**Authors:** Georg Gramse, Andreas Schönhals, Ferry Kienberger

**Affiliations:** a Johannes Kepler University , Biophysics Institute , Gruberstr. 40 , 4020 Linz , Austria . Email: georg.gramse@jku.at; b Bundesansatlt für Materialforschung und -prüfung (BAM) , Unter den Eichen 87 , 12205 Berlin , Germany; c Keysight Laboratories , Keysight Technologies , Gruberstr. 40 , 4020 Linz , Austria

## Abstract

We investigate the nearfield dipole mobility of protein membranes in a wide frequency range from 3 kHz to 10 GHz. The results of our nanoscale dielectric images and spectra of bacteriorhodopsin (bR) reveal Debye relaxations with time constants of *τ* ∼ 2 ns and *τ* ∼ 100 ns being characteristic of the dipole moments of the bR retinal and α-helices, respectively. However, the dipole mobility and therefore the protein biophysical function depend critically on the amount of surface water surrounding the protein, and the characteristic mobility in the secondary structure is only observed for humidity levels <30%. Our results have been achieved by adding the frequency as a second fundamental dimension to quantitative dielectric microscopy. The key elements for the success of this advanced technique are the employed heterodyne detection scheme, the broadband electrical signal source, a high frequency optimized cabling, development of calibration procedures and precise finite element modelling. Our study demonstrates the exciting possibilities of broadband dielectric microscopy for the investigation of dynamic processes in cell bioelectricity at the individual molecular level. Furthermore, the technique may shed light on local dynamic processes in related materials science applications like semiconductor research or nano-electronics.

## Abstract 2

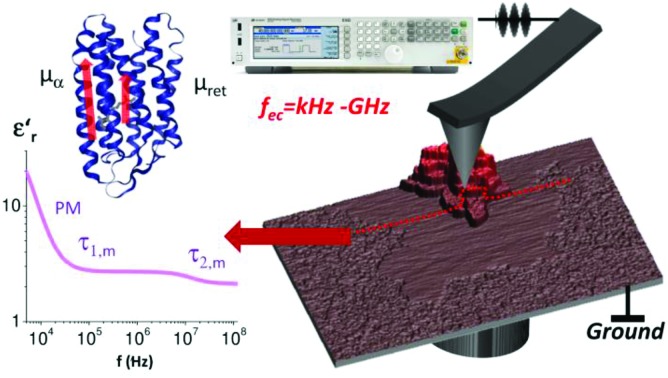
We investigate the nanoscale dipole mobility of proteins in a wide frequency range from 3 kHz to 10 GHz by broadband dielectric microscopy and reveal characteristic Debye relaxations.

## Introduction

The dielectric permittivity of membranes is important for many fundamental electrophysiological functions like selective transport in ion channels,^1^ action potential propagation^2^,^3^ and energy generation.^4^,^5^ The frequency dependency of this property plays hereby an essential role, since it determines how fast the dipoles associated with the integral molecular protein structures change the conformation according to an external electrical field. The mobility of the dipoles is strongly coupled with the presence of bound water which on the one hand enables dipole movements and the function of the protein itself.^6^ On the other hand, the outstanding dielectric properties of water itself are a very active field of basic research.^7^

Despite the importance of the dipole dynamics for the biophysical function of the protein, the intrinsic time constants in which structural changes in proteins occur can be used also as a dielectric fingerprint. At THz and higher frequencies, the identification of specific materials by their “dielectric” or “chemical” fingerprint has been proven to be successful for applications ranging from semiconductors^8^ to viruses.^9^

However, dielectric fingerprinting and investigation of nanoscale dielectric relaxation and resonant processes at lower time scales are currently not accessible.

Established techniques like dielectric^10^–^12^ or impedance spectroscopy^13^,^14^ provide precise permittivity spectra, however, only with a low spatial resolution down to a few μm, at best.^10^ This limits severely the investigation of many biophysical processes that take place at the nanometre scale. Moreover, samples might not be available in larger quantities or cannot be used in large ensemble measurements.

Electrical scanning probe microscopy techniques like Electrostatic Force Microscopy (EFM) offer superior spatial resolution down to the nm scale^15^–^18^ and provide quantitative information on the dielectric properties of a wide range of materials in both dry and liquid environments.^17^,^19^–^23^ Also the molecular growth of water films on flat substrates was investigated through the dielectric contrast.^19^,^24^,^25^ However, the assessment of the molecular dynamics and thus the frequency dependent dielectric properties of bio-membranes at the nanoscale has not been achieved yet. Here we study at the nanoscale the dielectric response of a protein membrane called bacteriorhodopsin (bR) that is found in the purple membrane (pm) of *Halobacterium salinarum*. It acts as a light-driven proton pump creating a proton gradient for energy generation in the cell (see ESI1 and Fig. S1†).

Because of its extraordinary functionality bacteriorhodopsin is of high scientific interest. Moreover, several high-tech applications based on bacteriorhodopsin, for instance high capacity data storage, are suggested.^26^

**Fig. 1:**
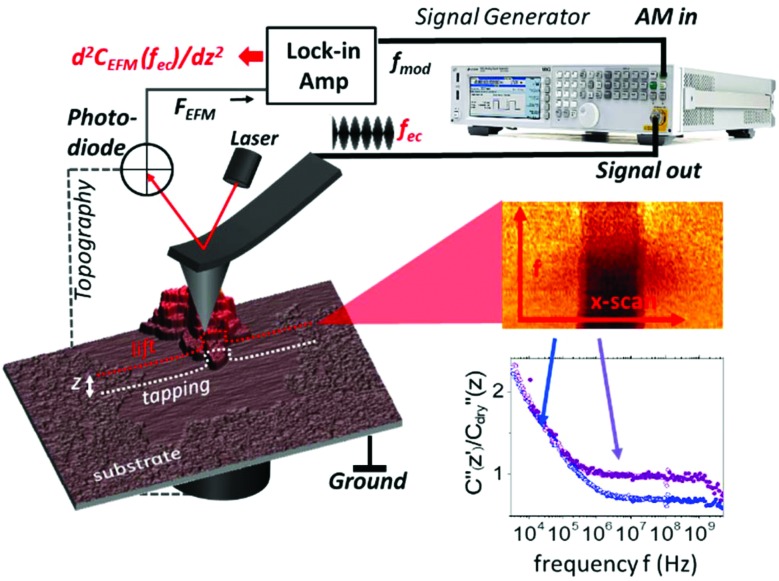
Experimental setup of a broadband impedance microscope. A signal generator delivers a frequency modulated signal to the AFM probe with a modulation frequency of 1.2 kHz and a variable carrier frequency between 3 kHz and 10 GHz. The induced electrostatic force is recovered through the lock-in amplifier by phase detection. Electrostatic force spectra are acquired in lift mode at a height *z* above the last topography scanline. To acquire dielectric spectra the slow scan is stopped and the frequency *f*_ec_ is changed every scanline. ∂*C*^2^/∂*z*^2^ (2π*f*_ec_) scanlines are averaged and post-processed to obtain dielectric spectra on the substrate and the membrane. Typically the dielectric spectra (3 kHz–10 GHz) acquired on the humid substrate (blue) and purple membrane (purple) are shown at the bottom right.

On flat substrates the membranes form a two dimensional lattice with a high density of protein that remains functional even after drying (see ESI1†).^27^ Nevertheless, the water environment plays an essential role in dipole fluctuations in the protein as well as the stacking of proteins into layers. To assess these properties at the nanoscale the EFM technique is used here that exploits the high force sensitivity of the AFM cantilever to detect the extremely small changes of the electrostatic force 
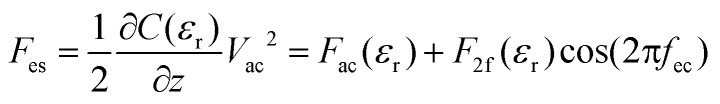
 induced by applying an external alternating voltage of *V*_ac_ = *V*_ec_cos(2π*f*_ec_) with a frequency *f*_ec_ between the tip and the sample. To investigate *ε*_r_ in a wide frequency range above and below the cantilever resonance frequency we augmented the standard EFM setup as shown in Fig. 1. In contrast to conventional EFM where *F*_2f_ is measured, in the set-up used here the down-modulated electrostatic force *F*_ac_ = 1/4∂*C*/∂*z*|*V*|_ec_^2^ is sensed.^21^,^28^–^30^ To increase the sensitivity the excitation signal is amplitude modulated with a low frequency, chosen to be conveniently detected by the cantilever. This enables the acquisition of the absolute part of the complex capacitance derivative *C*′(*z*, *ε*_r_, *f*_ec_) = |∂*C*/∂*z*|(*z*, *ε*_r_, *f*_ec_) at theoretically any excitation frequency. To improve further the lateral resolution we used phase detection^31^ and acquired the second order derivative of the capacitance *C*′′(*z*, *ε*_r_, *f*_ec_), which is related to the nanoscale complex dielectric permittivity *ε*_ρ_(*f*) = *ε*′_r_(*f*) + *jε*′′_r_(*f*) of the sample under study. A systematic quantification procedure, based on finite element modelling, is used to extract the molecule dynamics from the nanoscale dielectric measurements. The focus on dielectric properties in biophysics and the wide frequency range differentiate the used approach from current developments in the field of dynamic KFM techniques.^32^,^33^

## Results and discussion

**Fig. 2:**
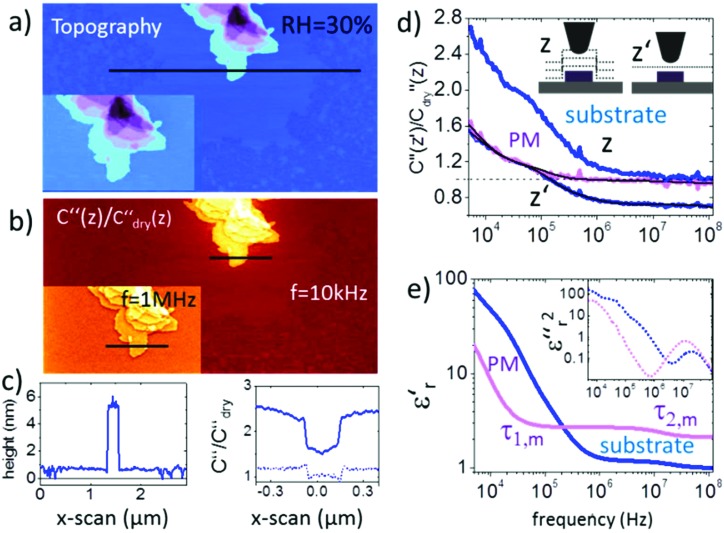
(a) AFM topography and (b) corresponding *C*′′(*z*)/*C*′′_dry_(*z*) image obtained in lift mode at *z* = 10 nm above the last scan line and at a frequency of *ω* = 10 kHz (inset at 1 MHz). The corresponding topography and *C*′′(*z*)/*C*′′_dry_(*z*) profile lines are shown in (c). Solid lines correspond to profile lines at 10 kHz and the dashed line to 1 MHz. (d) Normalized dielectric spectra on the substrate and protein membrane at constant height *z*′ = 15 nm and lift mode *z* = 15 nm. Black solid lines represent fitting with eqn (1) and (2). (e). Resulting complex dielectric functions *ε*′_r_(*f*) and *ε*′′_r_(*f*)^2^ (using the relation *ε*′′_r_(*f*) = –(π/2∂)*ε*′_r_/∂ln(2π*f*)^38^). All measurements are carried out at 25 °C using PtSi-FM tips from NanoSensors (Germany). Humidity was changed and left to stabilize for 2–3 hours. Imaging conditions were adjusted to maintain the lift distance for the dielectric images identical.

Membrane patches from bacteria rhodopsin prepared on a flat mica surface were imaged at a relative humidity RH = 30% as shown in Fig. 2. The topography images in Fig. 2a display how the membrane patches stack up to mono-, bi- and multilayers with a height difference of ∼5 nm for each layer. The presence of pure water patches on the mica surface can be observed, whereas at low humidity no water is present and at high humidity the water film covers the entire mica substrate (as proven independently from the force distance curves and images in Fig. S2, S3 and ESI2†). The second order derivative of the capacitance images (Fig. 2b and inset) is acquired in lift mode at *f*_ec_ = 10 kHz and a lift height of 10 nm. The data are normalized by the second order derivative of the capacitance acquired for a dry mica substrate *C*′′_dry_ (RH < 5%) having a constant dielectric permittivity of *ε*_r_ ∼ 6.5 within the used frequency window. The resulting *C*′′/*C*′′_dry_ image and the corresponding profile line in Fig. 2c exhibit a dielectric contrast of *C*′′_RH30_/*C*′′_dry_ = 1.5 between the mica substrate and the purple membrane. When decreasing/increasing the humidity of the environment we observe a decrease/increase in the contrast to *C*′′_RH5_/*C*′′_dry_ = 0.95 and *C*′′_RH65_/*C*′′_dry_ = 2.1, respectively (see also Fig. S2 and S7†). The above observation is associated with the presence of surface water exhibiting a higher dielectric permittivity and is expected to be present on both the AFM probe and the sample. The presence of a water film on the substrate is slightly visible in Fig. 2b and we also observe a pronounced border effect on the monolayers with an ∼10% higher signal along the borders than in the central area of the monolayers which is neither visible in the topography channel nor correlated with the phase or amplitude image. This observation points to a stronger hydration of the membrane at the interface to the substrate. Interestingly, when changing the measurement frequency to *f*_ec_ = 1 MHz the overall *C*′′_RH30_/*C*′′_RH5sub_ signal decreases from 1.5 to 1 and also the contrast between purple membranes reduces to 0.1, which has to be associated with a change in the dielectric properties of the membrane.

To extract the frequency dependent dielectric permittivity the profile line in Fig. 2c was imaged at 128 logarithmically spaced frequency points from 3 kHz to 120 MHz.

Fig. 2d shows the normalized *C*′′_RH30_/*C*′′_RH5sub_ data acquired at RH = 30% (raw data are shown in Fig. S4†). Topographical crosstalk is removed by acquisition of several spectra at increasing lift heights of *z* = 10, 20, and 30 nm, from which the spectrum at constant height *z*′ is calculated as detailed in the Experimental section. Comparison of the spectra obtained on the substrate in lift mode and in constant height mode in Fig. 2d shows that the dielectric signal is strongly affected by the non-local topography crosstalk and its removal is essential for the subsequent quantitative analysis. The influence of topographic crosstalk has been extensively discussed in various studies.^34^–^37^ The normalized data are quantified using finite element modelling as discussed further in the Experimental section. In short, the measured electrostatic force *F*(*G*,*z*,*ε*_r_,*σ*,*f*) depends on the tip–sample geometry, *G*, and distance, *z*′, and the dielectric (*ε*_r_) and conductive (*σ*) properties of the sample. The sample geometry and distance are known from the topography and the set imaging parameters. The electrical tip geometry is extracted from *F*_es_(*z*) approach curves.^39^ With the above parameters fixed, the finite element model is simulated and the electrostatic force is calculated for a wide set of parameters *F*_FEM,G,z_(*ε*_r_,*σ*,*f*), which is then used as a transfer function to fit experimental dielectric spectra with a set of Debye relaxations. In a first step, the spectrum of the substrate containing only a thin layer of surface water is analyzed with eqn (1) containing three relaxation terms with the parameters Δ*ε*_r,w,*i*_ and *τ*_w,*i*_ being the dielectric strength and characteristic time of each relaxation term, respectively. *ε*_r,w,*∞*_ and *σ* denote the high frequency dielectric permittivity and dc conductivity, respectively.1
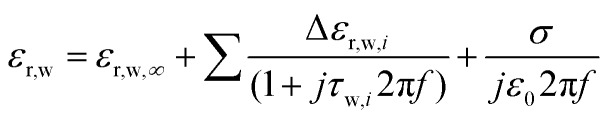



With these parameters fixed, eqn (2), containing additional relaxation terms, is fitted to the spectrum of the protein membrane. This approach assumes that the protein does not change the relaxation processes of the water at the substrate.2
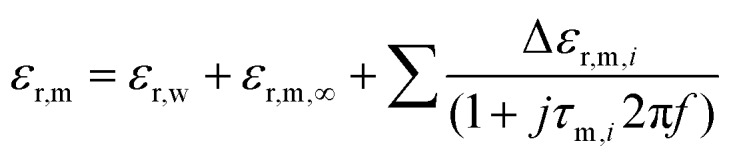



The two components of the complex dielectric function *ε*′_r_(*f*) and *ε*′′_r_(*f*) obtained by this analysis for the membranes on both the substrate and the bare substrate are shown in Fig. 2e (note that the loss term has been squared to amplify the relaxation processes). The parameters extracted in the fitting procedure are summarized in Table 1.

**Table 1:** Fitting results from dielectric relaxations under different humidity conditions in Fig. 2 and 3. The results are compared with the data from ensemble measurements by dielectric spectroscopy

	RH < 1%	RH = 35%	RH = 65%	Si/SiO_2_ RH = 30%	Ermolina *et al.*^40^
*ε*_r,m,*∞*_	2.7 ± 0.2	3.2 ± 0.2	3 ± 0.4	2.5 ± 0.02	6.8
Δ*ε*_r,m,1_	—	19 ± 5	140 ± 82	—	0.4
Δ*ε*_r,m,2_	—	0.5 ± 0.1	0.1 ± 0.07	0.5 ± 0.2	0.03
Δ*ε*_r,m,3_	—	0.08 ± 0.03	—	0.13 ± 0.03	0.1
*τ*_1,m_ (ms)	—	1 ± 0.5	0.6 ± 0.3	—	0.1–1
*τ*_2,m_ (ns)	—	100 ± 20	100 ± 140	150 ± 25	180–220
*τ*_3,m_ (ns)	—	1.5 ± 2	—	2.1 ± 1.4	2–20

The relaxations observed for the bare substrate are dominated by the dc-conductivity and non-local electrode polarization effects of the substrate and AFM probe. They will not be discussed in detail here. For the purple membrane two relaxation processes are extracted in the ms and sub-μs range. A further third relaxation process was obtained at about 1 ns by extending the frequency range to 10 GHz (see the spectrum in Fig. 1 and Fig. S6†).

Importantly, these relaxations for the membrane are not observed when the spectrum is acquired in a dry environment. In this case only a constant value *ε*_r,m,*∞*_ ∼ 3 is observed in agreement with the results from Dols *et al.*^39^ At a higher humidity of RH = 60% similar relaxation times to those for RH = 35% are extracted (spectra are shown in Fig. S7†).

**Fig. 3:**
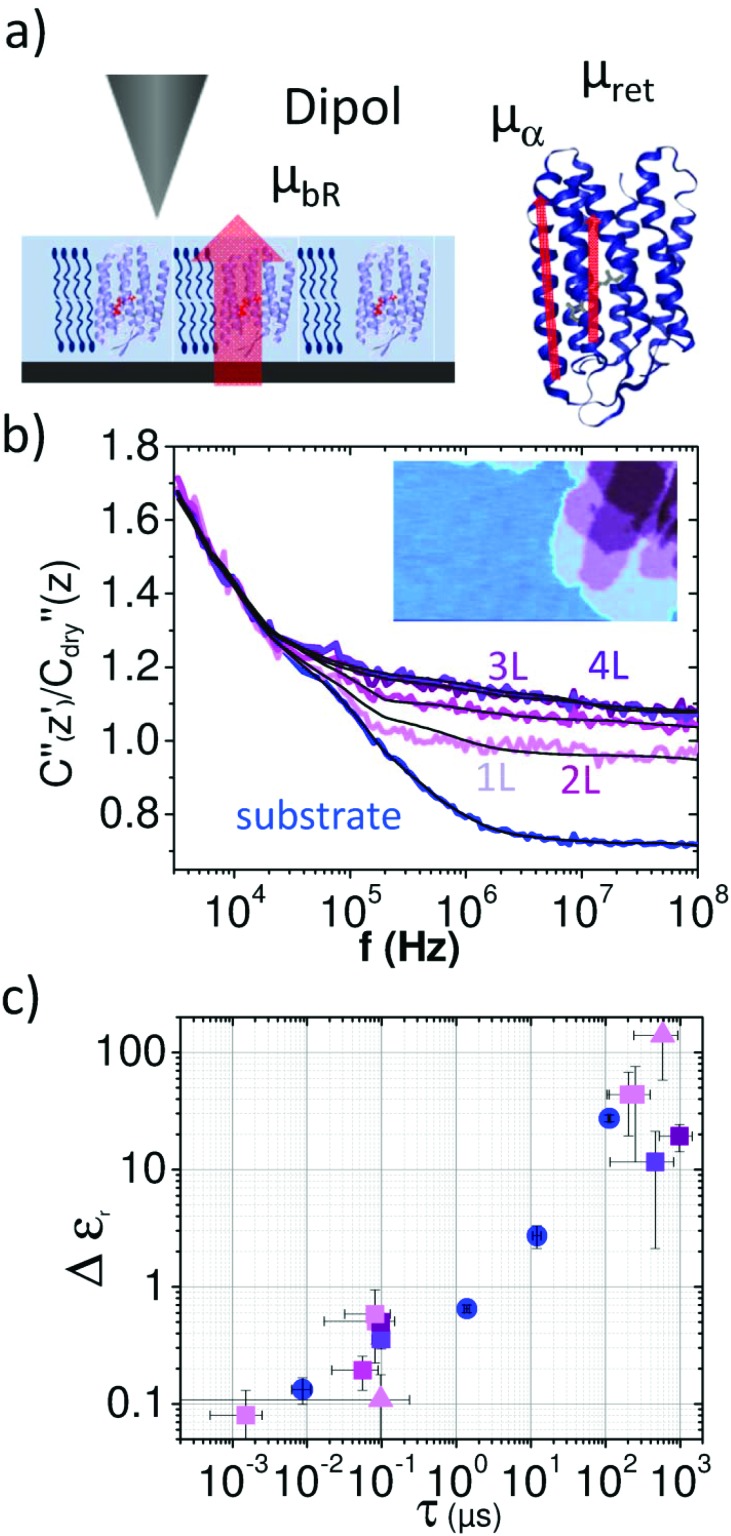
(a) Normalized dielectric spectra on the substrate (blue) and on single layer 1L, bi-layer 2L, triple layer 3L and four layer 4L of the purple membrane. Solid black lines represent fitting with eqn (1) and (2). (b) Measured force spectra are associated with molecular BR dipole movements. The inset shows the topography image of the region where spectra were acquired. (c) Δ*ε*_r_*versus τ* of the fitted relaxations displayed in the same colors (circles denote the substrate, squares the membranes). Also relaxations on the purple membrane from (Fig. 2e and Fig. S6†) are included (triangles).

Considering the extremely small amount of proteins that have been sensed by the nanometric probe the relaxation times of the two faster processes agree surprisingly well with the values from ensemble measurements obtained by Ermolina *et al.*^40^ Even the dielectric strengths for these two processes of our measurements agree by an order of magnitude with the literature values.^40^ According to the dipole moments the time constants can be related to the entire molecule movement (*τ*_1,m_), helical fluctuations of the α-helices (*τ*_2,m_) and the anisotropic movement of the retinal (*τ*_3,m_), respectively^40^ as sketched in Fig. 3a (see also ESI1 and Fig. S1†).

In Fig. 3b we compare the dielectric spectra obtained for a single layer and multilayers of the purple membrane. The inset shows the topographical image of the layers where the spectra were acquired. While for the single layer (1L) and the bilayer (2L) the difference in the signal is significant, the measurements for triple (3L) and four layers (4L) are almost identical. The results of the fitting procedure are summarized in Fig. 3c where the obtained relaxation times are plotted *versus* Δ*ε*_r,w,1_ (blue) and Δ*ε*_r,m,1_ (purple), respectively. Also, the values from Table 1 are included. We observe that the results of all experiments cluster at relaxation times of around *τ*_1_ = 100 ns and Δ*ε*_r,m_ = 0.5 well separated from the relaxation events of the surface water at ∼0.01 μs, ∼1 μs and ∼10 μs (*cf.*Fig. 3c blue circles). In contrast, the relaxation processes between *τ*_2_ = 0.2 and 1 ms are not as clearly separated from the water relaxation at about *τ*_w,4_ = 0.1 ms. For the multilayer we find a significantly lower dielectric strength for the first relaxation process.

Since our measurements are carried out at the nanoscale, we find important differences in our results, compared to ensemble measurements: firstly, at the nanoscale we measure by a factor of two a lower value of *ε*_r,m,*∞*_ both in dry^39^ and humid environments compared to ensemble measurements.^40^ Secondly, we note a clearly higher dielectric strength for the slowest process compared to the data given in reference 40. Furthermore the dielectric strength of this process drops with humidity. This might be due to the fact that at the nanoscale, we detect the local presence of surface water on the membranes which governs with its high dielectric permittivity the signal and therefore increases the apparent dielectric strength. On the other hand, a higher mobility of dipoles is expected for a thicker water film. Additionally it is also possible that for *τ*_1_ an electrode polarization process is observed, which with the additional interface of the purple membrane shows a different dielectric strength and is therefore not subtracted.

**Fig. 4:**
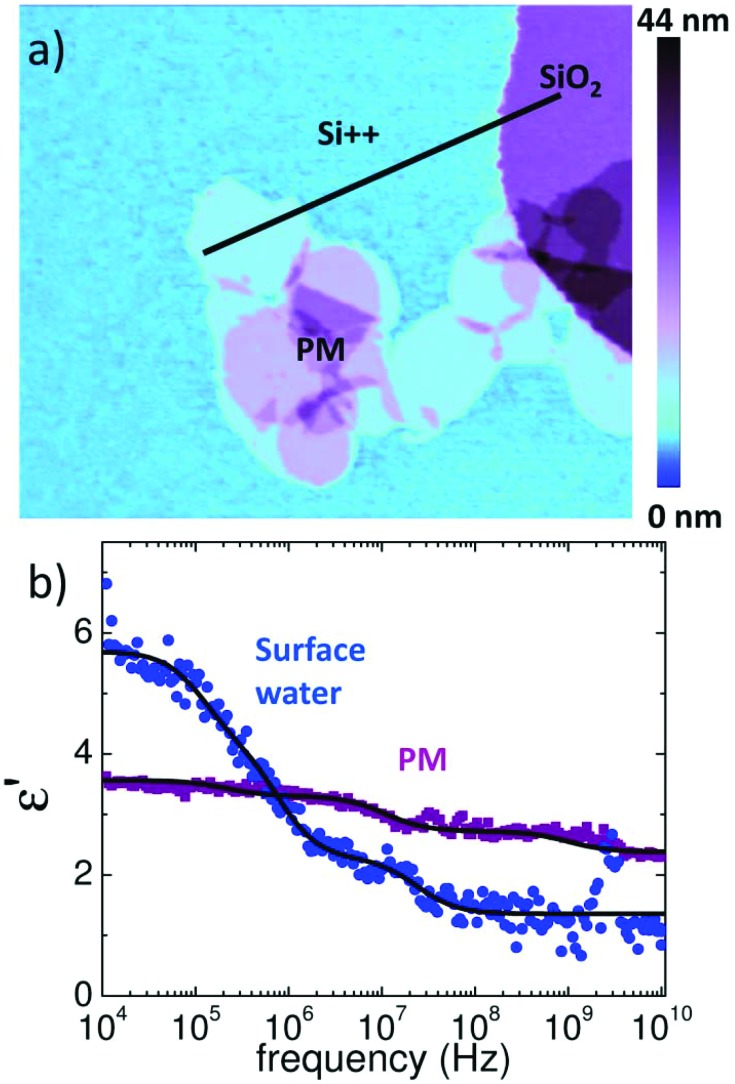
Dielectric spectra of PM on a highly doped Si/SiO_2_ substrate at RH = 30%. (a) Topography image of single- and multilayers of PM on the Si substrate. A 20 nm large SiO_2_ structure is visible on the top right. (b) The dielectric spectrum of surface water acquired on Si/SiO_2_ is shown in blue, and the spectrum of PM is shown in purple. The black line marks the position where spectra were acquired. Debye relaxations are fitted to the data (black solid lines). The apex radius was 55 nm and lift heights were 20 nm, 30 nm, and 50 nm. Further details are given in the text.

To clarify this point we repeated the measurements on a highly doped Si substrate (*ρ* = 0.005 Ω cm) which virtually acts as a ground. The bare Si exhibits additionally 20 nm high posts of SiO_2_ that act as a reference for the extraction of the dielectric constant. A topographic image of the PM sample is shown in Fig. 4a. The spectra shown in Fig. 4b were acquired at RH = 30% on the monolayer of PM, Si++ and SiO_2_. Data were normalized as on mica, however, in the final step the finite element simulations were used to convert normalized force data directly into *ε*′_r_. Note that the finite element model was different from that for mica as we detail in the Experimental section. By using SiO_2_ as a reference with known *ε*_r_ = 3.8 we extract directly from the forces, *F*_SiO2_ and *F*_Si_, acquired on Si and SiO_2_, respectively, the dielectric spectrum of the surface water, which is fitted as on mica with a sum of three Debye relaxations (eqn (1)). Finally we apply eqn (2) to extract the spectrum on PM. The result of the fitting procedure on PM is summarized in Table 1 and reveals that the first process has almost completely ended or at least is not detectible anymore at 10 kHz. This supports the hypothesis that the slower time constant *τ*_1_ is rather an electrode polarization effect, which can be much better observed on mica where the surface water is clearly more conductive due to the dissolved ions from the mica substrate.^41^ On Si in contrast we expect a much lower ion conductivity of the surface water, since no ions are present that can dissolve.

Similarly as on mica, also on Si we find the two faster PM relaxations *τ*_2_ and *τ*_3_ at about 1 ns and 100 ns. Note that on a highly conductive Si substrate the dielectric contrast is increased and both relaxations are more clearly visible than on mica.

Finally we note that the faster processes associated with the protein's secondary structure are less affected by the humidity and mobility dies out only in a completely dry environment (RH < 5%) when the thin water cushion between the substrate and protein membrane disappears.

Although the here studied protein membrane bacteriorhodopsin remains bio-functional even in humid air,^27^ we still expect some minor differences in its dipole dynamics when measured in its natural salt solution environment. The main drawbacks of the here presented approach with respect to in-solution measurements are certainly the missing fine control of ionic strength of the surrounding water film and the fact that the protein membranes are supported by a substrate. Additionally, we note that many other proteins remain dysfunctional when they are removed from the liquid environment, and therefore measurements on proteins should be carried out in solution, which will be the scope of future studies. Nevertheless many other relevant biomolecules like DNA or viruses/bacteria can be studied in a humid environment.

## Experimental

### Sample preparation

Bacteriorhodopsin was ordered from Sigma Aldrich (Germany) and diluted to 5 μg ml^–1^ in MilliQ water. An ultra-flat mica substrate was freshly cleaved and incubated with the diluted bR-solution for 10 min. A dry nitrogen stream, applied perpendicular to the sample, was used to carefully blow-off the remaining solution from the mica. This procedure produced single layer and multi-layer membrane patches of >500 nm in diameter.^30^ The same procedure was repeated for Si substrates.

### Experimental setup

The experimental setup for broad band dielectric microscopy is shown in Fig. 1 and consists of a 5600 AFM (Keysight Technologies, USA) interfaced with a signal generator through a coaxial cable and tip holder capable of delivering high frequency signals up to the tip (SMM nose cone). We used a MXG5183B analogue signal generator to cover a frequency range from 10 kHz to 10 GHz and a 33622A waveform generator to cover the range from 3 kHz to 120 MHz. A sinusoidal voltage signal with an angular frequency *f*_ec_ (carrier) is applied between a conductive probe and the bottom of the sample. When the carrier frequency is beyond the mechanical resonance of the cantilever it still bends the cantilever in a static way due to the non-linear dependence of the actuation force on the applied voltage. The amplitude modulation of the carrier at a low frequency, *f*_mod_, of about 1 kHz leads to an oscillation of the down-modulated force, which is detected with an enhanced S/N ratio using a lock-in amplifier^28^ sensing the phase shift of the mechanical cantilever oscillation at *f*_mod_.

EFM phase shift images and spectra are acquired in lift mode at distances >10 nm to exclude mechanical crosstalk and other short range interactions. Nonlocal topographical crosstalk is removed by acquisition of dielectric spectra for at least three lift distances above the surface. By interpolation between these distances we convert the lift-mode images into constant height images, which can be fitted as discussed in the next section and the main text. Similar approaches have been reported to remove non-local crosstalk effectively.^34^–^36^

### Finite element modelling

Finite element modelling was carried out with Comsol Multiphysics 5.2 (2D axisymmetric, AC/DC module, electric currents, frequency domain). The simulations for quantification resemble the experimental conditions and are sketched in Fig. S3.† The model consists of a 15 μm high AFM tip modelled as a truncated cone with a cone angle *θ* and a cantilever extending the cone end by 10 μm (not fully shown in Fig. S5†). The tip has a spherical apex with a radius *r*_a_ and is located at a distance *z* above the sample. The sample comprises the *h* nm thick membrane with the dielectric permittivity *ε*_r,m_ and the conductivity *σ*_m_ on the mica substrate.^39^,^42^ The substrate itself is assumed to be a perfect dielectric with *ε*_r_ = 6.5 (mica) and *ε*_r_ = 3.8 (SiO_2_). Both the mica substrate and probe-surrounding air are of infinite size in both *r* and *z* directions (infinite elements are used on outer boundaries).^42^ The highly doped Si substrate (*ρ* = 0.005 Ω cm) is considered to act as a ground. To achieve accurate modelling results, meshing was set to scale with a size of the involved geometries. In this way the mesh size on the boundary of the apex and membrane was always at least 20 times smaller than that on the tip apex. The model was meshed from the inside (apex + membrane) to the outside (cone + infinite elements). Outside parts were meshed automatically by Comsol. The downward directed Maxwell-Stress-Tensor in the *z* direction was calculated on the tip boundaries which had been set to Terminal 1V. From these data the second order derivative of the capacitance was calculated. To determine the tip-geometry a combination of experiments and simulations was carried out. In detail, electrostatic force approach curves (*z* from 2–500 nm) were simulated for a wide range of tip radii, *R*, and cone angles, *θ*. Using a fitting procedure with the simulated approach curves, the tip radius and cone angle are extracted that leads to the best agreement with the experimental approach curve.^42^ After the estimation of the tip geometry the electrostatic force *F*_es_(*z*,*h*,*σ*,*ε*_r_,*f*) ∼ d*C*^2^/d*z*^2^(*z*,*h*,*σ*,*ε*_r_,*f*) was calculated for a wide range of dielectric parameters (*σ*,*ε*_r_) keeping the geometry fixed and a lookup table/interpolation function was generated from these data.^30^,^32^ The tip sample distance, *z*, the sample thickness, *h*, and the measurement frequency, *f*, are known set parameters. For the conductivity *σ* and dielectric permittivity *ε*_r_ we use the real and imaginary parts of eqn (1) and/or (2), respectively, and fit the Debye model to the data.

## Conclusions

The extraction of nanoscale dielectric spectra from single and multilayer protein membranes by broadband dielectric microscopy has been demonstrated and relaxation times of *τ* ∼ 100 ns and *τ* ∼ 10 ns associated with the molecular protein dipole moments have been estimated. The technique adds information on the local dynamics of dipoles and charges in single layered biological membranes and their interplay with the water environment. We found by measurements under controlled humidity conditions that the presence of surface water is essential to observe dipole mobility and associated dielectric relaxation events. The extracted relaxation frequencies characteristic of the studied protein can be used as a dielectric fingerprint. We achieved these results by combining the high spatial resolution and electrical sensitivity of EFM with the wide frequency range of a signal generator and appropriate cabling designed to deliver signals from kHz up to the microwave frequency range (10 GHz) to the end of the tip. Electrical sensitivities down to the zepto Farad level and nanometre resolution are the major advantages of this technique compared to ensemble measurements. Precise finite element modelling based quantification workflows have been established to allow for quantifiable interpretation of the results.

The developed procedures are extendible to biological systems operating in a liquid environment.^20^,^21^,^43^ A future scope will be therefore the investigation of voltage gated ion channels, which might shed light on fast dynamics in gating processes and charge transport relevant also for pharmaceutical applications. In these systems especially the frequency range from 10 MHz to 10 GHz will be of major importance, since it is not affected by ionic screening.^20^,^21^,^43^ Furthermore, the technique will be used to address the nanoscale conductive properties in semiconductor devices^44^ and of 2D materials at their typical operation frequencies.

## Conflicts of interest

There are no conflicts to declare.

## Supplementary Material

Supplementary information
