# Supplementary material for: Nanoscale dipole dynamics of protein membranes studied by broadband dielectric microscopy
Source: Nanoscale. 2019 Feb 5;11(10):4303–9. doi: 10.1039/c8nr05880f (PMC6457197; doi:10.1039/c8nr05880f)
Supplement: Supplementary file 1 [file NR-011-C8NR05880F-s001.pdf]

Electronic Supplementary Material (ESI) for Nanoscale.
This journal is © The Royal Society of Chemistry 2019

Supplementary Information

Nanoscale dipole dynamics of protein membranes by Broadband Dielectric Microscopy

G. Gramse[a,*] , A. Schönhals[b] and F. Kienberger[c]

[a.] *Johannes Kepler University, Biophysics Institute, Gruberstr. 40, 4020 Linz, Austria*
[b.] *Bundesansatlt für Materialforschung und -prüfung (BAM), Unter den Eichen 87, 12205 Berlin, Germany*
[c.] *Keysight Laboratories, Keysight Technologies, Gruberstr. 40, 4020 Linz, Austria.*
* Correspondence to: georg.gramse@jku.at

SI 1 Bacteria rhodopsin – Structure and Dipole moments

Bacteriorhodopsin is an integral membrane protein of the cell membrane of the organism *Halobacterium salinarum*. It acts as a light-driven proton pump creating a proton gradient between the interior of the bacteria (cytoplasm) and its exterior. This proton gradient serves as energy source for the ATP synthase [1-3].

The protein of bacteriorhodopsin consists of 248 amino acids (primary structure). These amino acids are arranged in seven nearly parallel α-helices (secondary structure). The helices percolate the cell membrane forming pores. Within a pore there is a retinal molecule bounded to the protein by an amid bond (Schiffsche base, amine function of the amino acid Lys216). The retinal molecule is the chromophore of the protein. Under physiological conditions it exist only as all-trans and 13-cis isomer. The isomerization is triggered by light. [4]

In the cell membrane bacteria rhodopsin is organized in trimers which are self-assembled in a hexagonal two-dimensional arrangement in the lipid double layer ordered forming two dimensional crystalline regions with sizes up to 5 micrometers. These crystalline regions are called purple membranes. This crystalline structure gives bacteria rhodopsin an extremely stability with regard to physical or chemical impacts. This means bacteria rhodopsin retains its color and photochemical activity even in the dry state and under the influence of oxygen [5].

The α-helix is a common secondary structure of a protein. The α-helix is the most stable natural conformation of a sequence of amino acids. Concerning the dipole moments of bacteriorhodopsin, one has to consider firstly that that the dipole moment of a peptide bond forming the protein is 3.5 Debye (see Figure S1). In the helical state of the protein all dipole moments of the peptide bonds are oriented in the same direction in parallel to the long axis of the helix. Therefore, the helix has a kind of super dipole n*2.5 Debye, where n is the number of the amino acids in the helix (see Figure S1). By dielectric spectroscopy the molecular fluctuations of the helical structure can be detected. Secondly, one has further to consider that the retinal molecule, which is not part of the helix carries also a dipole moment. For that reason, at least two dielectric relaxation processes one related to collective helical fluctuations

of the whole protein, the other one assigned to molecular fluctuations of the retinal molecule should be observed by dielectric spectroscopy. [6]

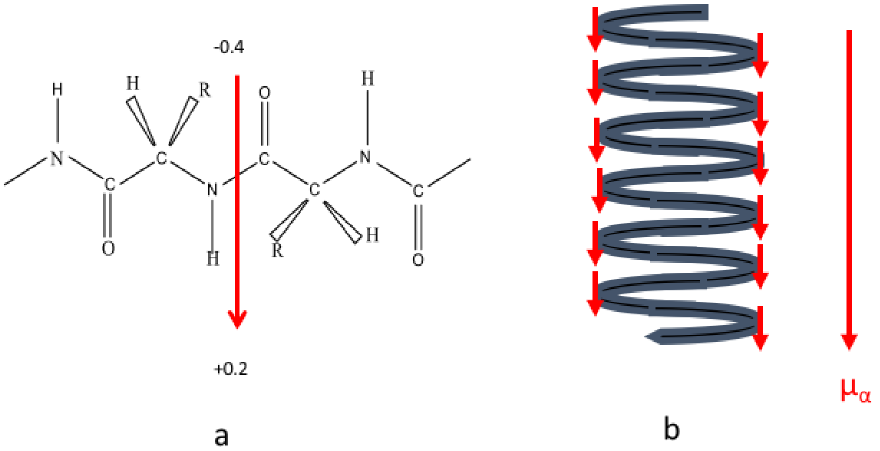

Figure S1. (a) Peptide bond including the corresponding dipole moment and (b) schematic protein matrix including the dipole moment in parallel to the long axis of the helix.

SI 2 Visualization of surface water from topography images and force distance curves

Fig. S2 shows a topography image of the purple membrane patch on the mica surface for different humidities values. The sample was left for drying for more than 6 hours at RH<5%. The substrate in Fig. S2a appears to be completely flat and a force versus distance curve shows only a small adhesion force of ~12nN. The humidity in the environment was increased to 30% and stabilized for 2 hours. The topography image reveals water patches of approximately 0.5nm in height. At RH=65% the topography appears to be flat again but force versus distance curves show a clearly higher adhesion force of 17nN.

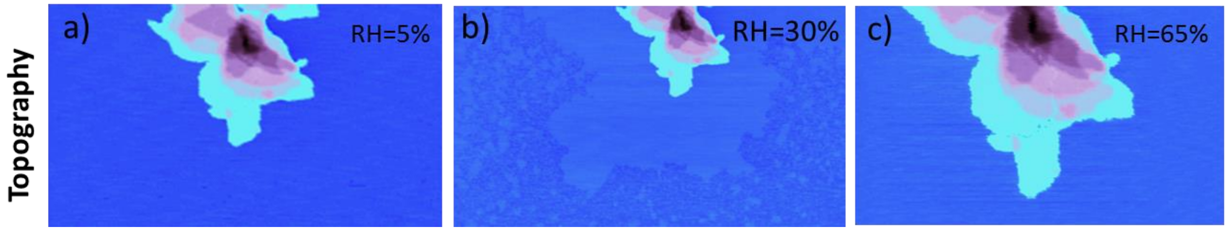

Figure S2. Topography images of purple membrane on mica with increasing values of relative humidity (RH).

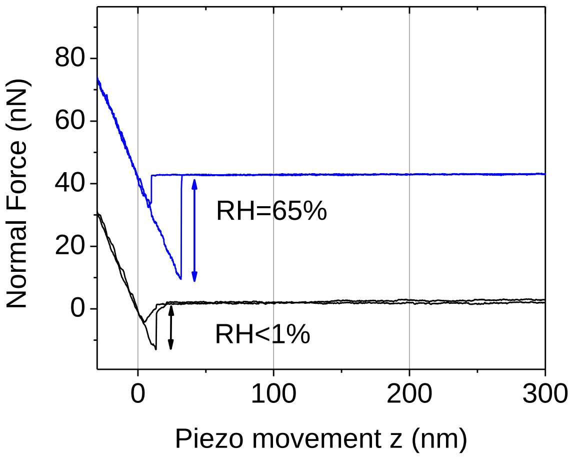

Figure S3. Force versus distance curves on mica at 5% humidity and 65% humidity. Curves are offset for sake of clarity.

SI 3 Dielectric Spectra on purple membrane: raw data

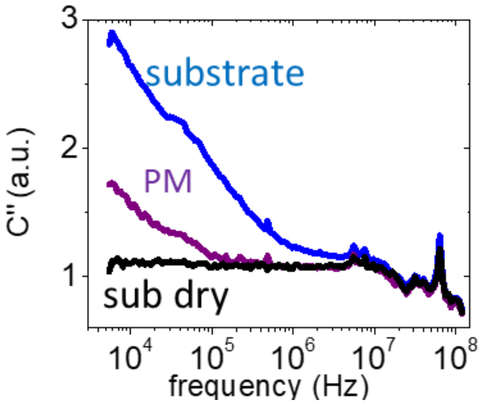

Figure S4. Dielectric spectra acquired on dry substrate (black), RH=30% substrate (blue), RH=30% purple membrane (purple). Spectra are shown without normalization. For frequencies above 10MHz reflections and loss in the cable have to be considered.

SI 4 Finite Element Modelling (FEM) for quantification of measurements

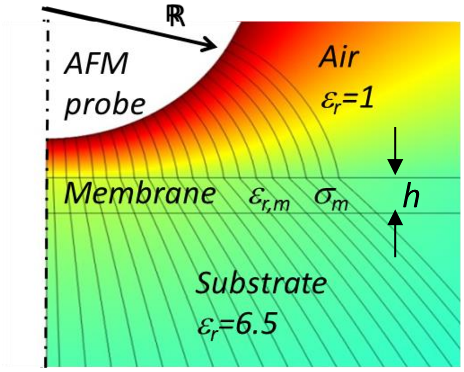

Figure S5. Finite Element Model used to quantitatively extract the frequency dependent dielectric data. A zoom in of the tip sample region is show. The important material/geometric parameters for the quantification are included.

SI 5 Dielectric spectrum with extended frequency range

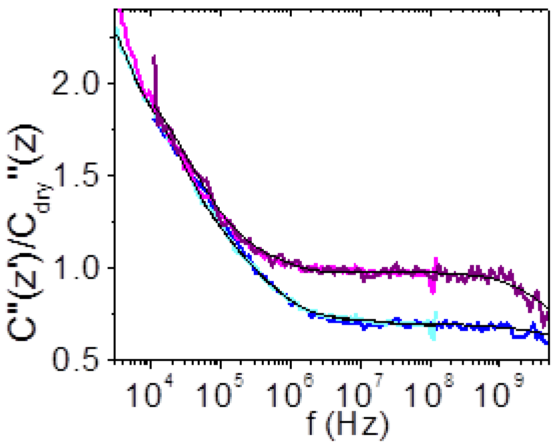

Figure S6 Dielectric spectrum from 3kHz to 10 GHz the purple membrane (purple) and the bare substrate (blue) at RH=30%. Solid lines represent best fit of the simulations to the data. Fitting results are summarized in Table 1 in the main text. Two signal generators were used to cover the entire frequency range. The frequency range from 3kHz to 120MHz was covered by the waveform generator 33620A from Keysight and the range from 10kHz to 10GHz was covered by an analog signal generator MXG5183B (Keysight, CA). The lift height was z'=10nm.

SI 6 Comparison of dielectric spectra on monolayer purple membrane at three values of the relative humidity

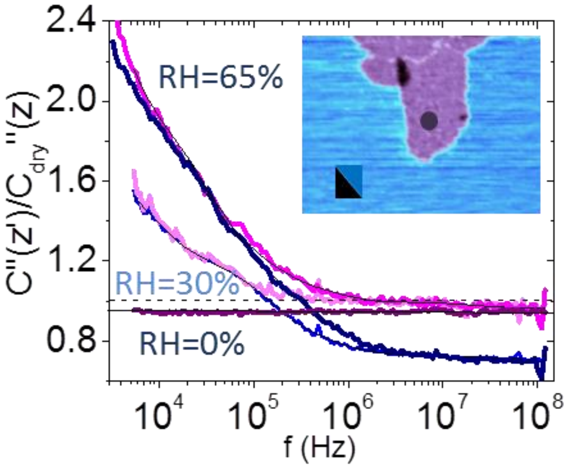

Figure S7 Comparison of dielectric spectra obtained at RH<5%, RH=30% and RH=65%. Spectra were obtained with the same probe at z'=10 nm. Blue lines correspond to data obtained ~~on~~ for the bare substrate; purple lines are data for the membrane, black lines correspond to fits with simulations. Fitting results are summarized in Table 1 (main text).

References

1. D. Oesterhelt and W. Stoeckenius, *Nature New Biology*, 1971, 233, 149.
2. D. J. Müller and A. Engel, *Nature Protocols*, 2007, 2, 2191.
3. T. Kouyama, K. Kinosita and A. Ikegami, *Advances in Biophysics*, 1988, 24, 123-175.
4. D.-l. Chen, G.-y. Wang, B. Xu and K.-s. Hu, *Journal of Photochemistry and Photobiology B: Biology*, 2002, 66, 188-194.
5. Y. Shen, C. R. Safinya, K. S. Liang, A. F. Ruppert and K. J. Rothschild, *Nature*, 1993, 366, 48.
6. I. Ermolina, A. Lewis and Y. Feldman, *J Phys Chem B*, 2003, 107, 14537-14544.
